# Diffuse melanosis cutis related to dermal micrometastases as the first clinical symptom of distant metastatic malignant melanoma: Case report

**DOI:** 10.1097/MD.0000000000006470

**Published:** 2017-04-14

**Authors:** Joanna Maj, Alina Jankowska-Konsur, Joanna Gruber, Zdzisław Woźniak, Piotr Nockowski, Anita Hryncewicz-Gwóźdź

**Affiliations:** a Department of Dermatology, Venereology and Allergology, Wroclaw Medical University; b Department of Pathomorphology, Wroclaw Medical University, Wroclaw, Poland.

**Keywords:** diffuse melanosis cutis, melanodermia, melanoma, melanuria, paraneoplastic syndrome

## Abstract

**Rationale:**

Diffuse melanosis cutis (DMC) is a very rare sign of malignant melanoma progression. The condition usually develops after approximately one year from melanoma diagnosis in a patient with metastatic tumors and after anticancer treatment with cytostatic medications.

**Patient concerns:**

A 72-year old Caucasian man was admitted to the Department of Dermatology with DMC for 4 months and the history of two melanomas treated surgically 30 years and 9 months before present hospitalization.

**Diagnosis:**

Histological and immunohistochemical examinations of DMC biopsy indicated melanoma metastatic cells as well as free deposits of melanin and melanophage presence in the dermis.

**Interventions:**

The patient refused to the treatment.

**Outcomes:**

The patient died eight months after DMC appeared.

**Lessons:**

DMC is a rare presentation of advanced MM and is a bad prognostic factor. The pathomechanisms of the discoloration of the skin are not fully explained. The role of micrometastases, as well as melanin precursors, released during lysis of MM metastases, and growth factors may play a role in the development of the symptom.

## 1 Introduction

The advanced stages of melanoma malignum (MM) can manifest with metastases into the lymph nodes, liver, lung, brain, bones, and skin.^[1]^ The skin and subcutaneous tissue metastases are relatively rare. They are usually located approximately 2 cm from the primary tumor and clinically present as small, bluish nodules. In rare cases, tumor progression manifests itself as a generalized discoloration of the skin which turns a blue–gray (diffuse melanosis cutis, DMC).^[2,3]^ The discoloration is usually more intensive on sun-exposed areas, including head and upper torso.^[4]^ Usually, DMC is associated with dark urine.^[5]^ Due to the rarity of the observed phenomenon the pathogenesis of DMC is yet to be fully understood. In the literature, approximately 70 cases of DMC have been described until now, most of them being associated with multiple organ metastases or chemotherapy treatment such as dacarbazine.^[3,6]^ The average time from MM diagnosis to the development of DMC is 1 year, and the survival time from the onset of this symptom is approximately 4–5 months.^[2]^ Herein, we present a case of DMC in the course of MM micrometastases to the skin. Due to MM, the patient underwent surgery twice, 30 years ago and 9 months before discoloration of the skin appeared. After the second operation, metastasis in the sentinel node was identified. DMC was the first sign of the extranodal progression of the tumor. No anticancer treatment was administered before DMC developed. Histological examinations indicated single-cell metastases (micrometastases) being involved in the development of skin discoloration.

## 2 Case presentation

**Figure 1:**
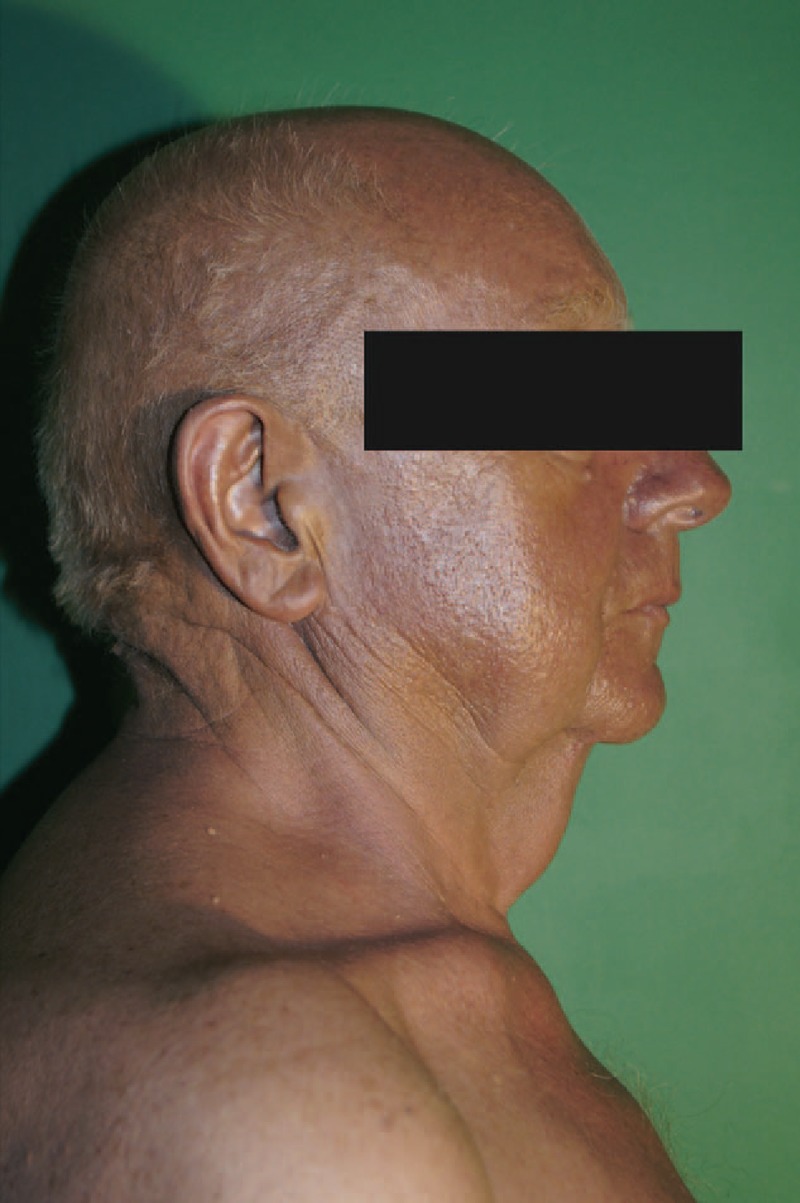
Dark–gray skin coloration on the face, neck and upper torso.

**Figure 2:**
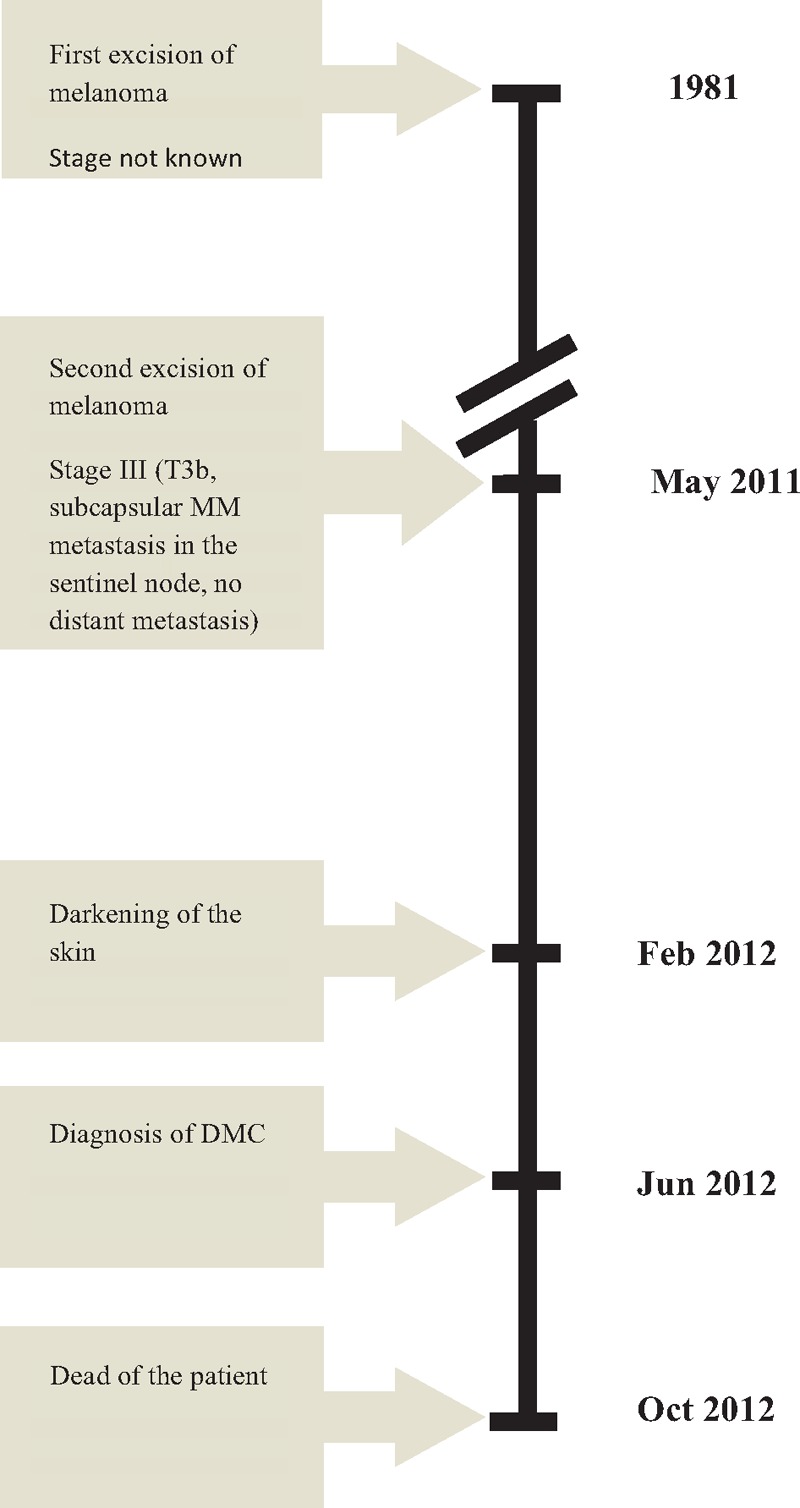
Timeline of most important events related to disease progression and diagnostic.

**Figure 3:**
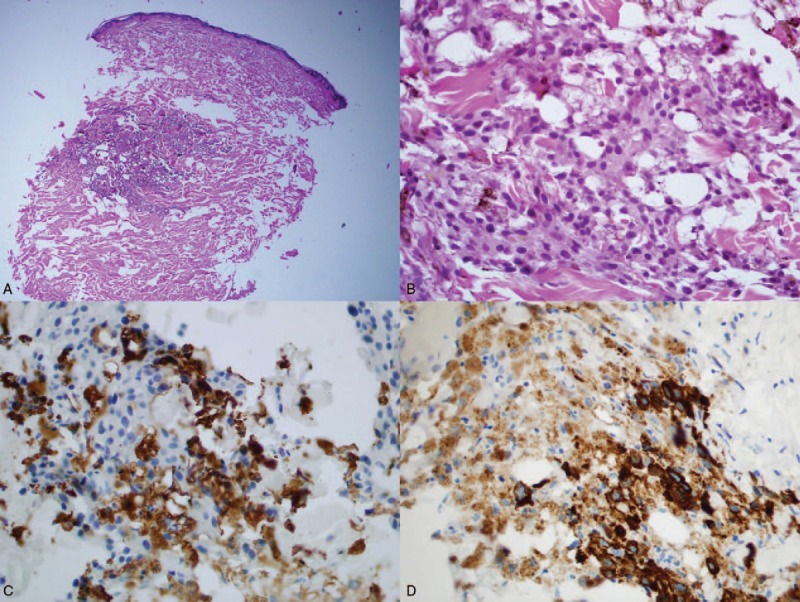
Histological examination of a skin biopsy (A—H&E staining ×100 and B—H&E staining ×400) performed in the region of clinically observed melanosis showed intracellular and extracellular melanin deposition and focal dermal infiltration by collection of pigment—laden macrophages (C—immunostain for CD68, ×400) and dermal melanoma metastatic cells (D—immunostain for HMB45, ×400).

A 72-year-old caucasian man suffering from angina pectoris, hypertension, type 2 diabetes, with a history of coronary angioplasty was admitted to the Department of Dermatology (Wroclaw Medical University, Poland) for diagnosis of a dark gray skin coloration which had been present for 4 months. The pigmentation intensity was most pronounced on the face, neck, and upper torso (Fig. 1). The patient reported general weakness, loss of appetite, weight loss, and dark urine for several weeks. Two weeks before admission to the Dermatology Department, the patient had been hospitalized in the Department of Internal Diseases, where other causes for the skin discoloration, that is, Addison's disease, and porphyria were excluded. The patient had a history of 2 surgeries due to MM (Fig. 2). In 1981 MM was located on the left thigh. The malignant lesion was removed; however, the patient did not keep the medical records considering the neoplasm staging. In 2011, a dark ulcerated tumor, 2 cm in diameter was removed from the left scapular region. Histological diagnosis of the latest lesion was nodular type MM, Clark IV, Breslow more than 3 mm. The left axillary nodes were removed. In the sentinel node, subcapsular MM metastasis was identified. Apart from surgery, no other oncological treatment was administered. Nine months after the second surgery, a generalized blue–gray discoloration of the skin appeared. Four months later, a diagnostic skin biopsy was taken from his right shoulder area and revealed intracellular and extracellular melanin deposition in the dermis. Intracellular deposition of melanin was detected in two types of cells: CD68(+) macrophages and melanoma metastatic cells with *immunohistochemical* profile: Melan-A (+), HMB45 (+), S100(+), CD68 (−), distributed and grouped into deep layers of the dermis (Fig. 3). Laboratory tests indicated slightly increased CA 15-3 antigen, beta-2-microglobulin, aspartate aminotransferase (AST), alanine aminotransferase (ALT), and triglycerides levels. The expression of S100 protein in serum was elevated: 19.320 μg/L (normal value < 0.105 μg/L). Urine analysis did not reveal any abnormalities other than its brown-amber color. Imaging tests (abdominal, thyroid and cardiac ultrasonography, chest x-ray and CT, colonoscopy, PET-CT) revealed a tumor of 2 cm in diameter, in the right kidney. The patient refused to the treatment and died eight months after DMC appeared. (Fig. 2)

## 3 Discussion

The usual location for distant metastases of MM comprises liver, lungs, brain and bones, as well as the skin and lymph nodes. DMC is a very rare manifestation of advanced MM. Until 2015, 70 cases of DMC were reported, however, the pathophysiological mechanisms of the condition have not been elucidated.^[2]^ So far, several alternative hypotheses concerning etiopathogenesis of DMC were proposed. Several lines of evidence suggest that the direct cause of the skin darkening is melanin derived from the melanoma micrometastases in the dermis.^[7,8]^ Other studies suggested that specific growth factors, including alpha-melanocyte-stimulating hormone (α-MSH), endothelin-1 and hepatocyte growth factor (HGF) released by melanoma cells could stimulate proliferation of normal melanocytes in the skin that would result in an increased number of melanocytes, melanogenesis, and skin pigmentation.^[9]^ On the other hand, other reports indicate that the main cause of the skin darkening is melanin precursors which are liberated into the bloodstream during the rapid lysis of MM metastatic tumors in the internal organs, a phenomenon triggered by anticancer therapy.^[3,6,10]^ Subsequently, in the dermis and urine melanin, precursors undergo spontaneous oxidation and are transformed into the melanin. This theory has been supported by the documented observations of melanin precursors, melanin, melanosomes, and melanophages in patients’ serum.^[3,6,11]^

On the contrary, in the presented case, only one small metastatic tumor was found in the kidney and chemotherapy was not applied. The release of melanin precursors from decayed metastatic mass, and due to its small size, could not have triggered diffuse melanosis. Therefore, we suggest that the main cause of DMC in our patient is the melanin produced by numerous dermal MM micrometastases.

## 4 Conclusion

DMC is a rare presentation of advanced MM and is a bad prognostic factor.^[1]^ The pathomechanisms of the diffuse skin discoloration are not fully explained. However, the role of micrometastases, as well as melanin precursors, released during lysis of MM metastases, and growth factors play a role. In our patient the tumor burden, which initiated the development of DMC, was not formed from clinically observed macrometastases like in most other cases, but from micrometastases to the skin. The extreme rarity of DMC in the course of metastatic melanoma, and in the case described above, the lack of macroscopic metastases caused a delay in DMC diagnosis for 4 months.
